# Genomic epidemiology of nontoxigenic *Corynebacterium diphtheriae* from King County, Washington State, USA between July 2018 and May 2019

**DOI:** 10.1099/mgen.0.000467

**Published:** 2020-12-04

**Authors:** Lingzi Xiaoli, Eileen Benoliel, Yanhui Peng, Janessa Aneke, Pamela K. Cassiday, Meagan Kay, Shelly McKeirnan, Jeffery S. Duchin, Vance Kawakami, Scott Lindquist, Anna M. Acosta, Chas DeBolt, Maria Lucia Tondella, Michael R. Weigand

**Affiliations:** ^1^​ IHRC, Inc., Atlanta, Georgia, USA; ^2^​ Public Health Seattle and King County, Seattle, WA, USA; ^3^​ Division of Bacterial Diseases, Centers for Disease Control and Prevention, Atlanta, GA, USA; ^4^​ Washington State Department of Health, Shoreline, WA, USA

**Keywords:** *C. diphtheriae*, genomics, nontoxigenic, SNP, virulence factor

## Abstract

Between July 2018 and May 2019, *Corynebacterium diphtheriae* was isolated from eight patients with non-respiratory infections, seven of whom experienced homelessness and had stayed at shelters in King County, WA, USA. All isolates were microbiologically identified as nontoxigenic *C. diphtheriae* biovar mitis. Whole-genome sequencing confirmed that all case isolates were genetically related, associated with sequence type 445 and differing by fewer than 24 single-nucleotide polymorphisms (SNPs). Compared to publicly available *C. diphtheriae* genomic data, these WA isolates formed a discrete cluster with SNP variation consistent with previously reported outbreaks. Virulence-related gene content variation within the highly related WA cluster isolates was also observed. These results indicated that genome characterization can readily support epidemiology of nontoxigenic *C. diphtheriae *.

**Outcome**

In this study, we investigated an outbreak cluster of nontoxigenic *C. diphtheriae* emerging in King County, WA, USA using whole-genome sequencing. Our genomic analyses confirmed clustering of these cases and revealed that *C. diphtheriae* can exhibit gene content variation during outbreak transmission. Thus, increased awareness of the risks for persistent nontoxigenic *C. diphtheriae* cutaneous infections among at-risk populations is needed.

## Data Summary

Genome sequence data have been deposited in GenBank, organized under BioProject accession number PRJNA541849. The complete genome assembly for PC0646 is available under accession number CP040557.

## Introduction

Diphtheria is characterized as toxin-mediated respiratory or cutaneous infections caused by toxigenic strains of *Corynebacterium diphtheriae* [1]. Following widespread application of effective vaccines against the diphtheria toxin, respiratory diphtheria cases have decreased significantly worldwide [2]. However, increased infections by nontoxigenic *C. diphtheriae* strains, which are not preventable by current vaccines targeting diphtheria toxin, have been reported [3–6]. Nontoxigenic *C. diphtheriae* infections often occur at cutaneous lesions but can progress to severe diseases such as bacteraemia, septic arthritis and endocarditis [3–6]. Population groups particularly at risk of *C. diphtheriae* infections include refugees [7], foreign travellers [8], intravenous drug users, or persons experiencing homelessness in metropolitan areas [3–6].

Molecular approaches developed for the surveillance and outbreak clustering of diphtheria can also inform epidemiology of *C. diphtheriae *. A multilocus sequence typing (MLST) scheme targeting housekeeping genes has been widely used for *C. diphtheriae* subtyping and currently includes more than 664 reported sequence types (STs) [9]. However, MLST provides limited resolution to study bacterial genetic relatedness, particularly within outbreaks that include case isolates of a single ST. MLST is also incapable of detecting gene content variations such as acquisition of certain antibiotic resistance genes and virulence factors. Alternatively, whole-genome sequencing (WGS) captures sequence variations across the full genome and thus can simultaneously be used to elucidate genetic links between cases and identify antibiotic resistance markers and virulence factors among isolates. Previous studies have successfully used whole-genome single nucleotide polymorphisms (SNPs) to confirm the clustering of isolates from diphtheria cases with known epidemiological or geographical associations [7, 10, 11]. Together, these reports suggest that isolates recovered from outbreak clusters of diphtheria frequently differ by fewer than 150 SNPs, whereas unlinked sporadic isolates differ by an average of 30 000 SNPs [7].

A few investigations of nontoxigenic *C. diphtheriae* have included WGS analyses, but the retrospective designs of those studies have been complicated by large temporal or geographical distributions among studied case isolates as well as sparse epidemiological data [12, 13]. Nontoxigenic *C. diphtheriae* is of public health concern due to the lack of protection provided by current diphtheria vaccines and the potential for such circulating strains to readily become toxigenic through lysogenization by toxin-encoding bacteriophages [14]. In the present study, we employed WGS to compare nontoxigenic *C. diphtheriae* isolates primarily recovered from skin lesions of epidemiologically linked cases in King County, WA, USA and quantify SNP accumulation during outbreak transmission.

## Methods

### Outbreak information

Between July 2018 and May 2019, 10 *C. diphtheriae* isolates were recovered from eight patients, seven of whom experienced homelessness and had stayed at shelters in King County, WA, USA. Patients were predominantly male (87.5 %), had a median age of 51.5 (range, 35–67) and had a documented history of intravenous drug (patients 2, 5 and 8) or methamphetamine abuse (patients 1, 4 and 7). These individuals registered stays at multiple homeless shelters in King County, including three in downtown Seattle (shelters A, B and C) separated by 0.3 miles and another (shelter D) approximately 2 miles away. The first *C. diphtheriae* case isolate (PC0646) was collected in July 2018, followed by seven cases reported between Jan 2019 and May 2019. Patients 1, 4, 5 and 6 registered overlapping visits at shelter A, ranging from 1 to 166 nights between October 2018 and April 2019, and all four patients were present in this shelter on one night. During investigation of the outbreak, a second report of *C. diphtheriae* infection was identified for two patients (patients 1 and 2), resulting in two isolates each from these individuals, while all other patients contributed one isolate to the study. Basic epidemiological case information is summarized in Table 1.

**Table 1.** Epidemiological and microbiological description of the WA *C. diphtheriae* isolates

Patient no.	Isolate	Collection date	Homeless	Location	Isolate source	MLST	Penicillin MIC
1	PC0647	Jan–2019	Yes	Shelter A	Leg wound	445	0.25
1	PC0650	Feb–2019	Yes	Shelter A	Leg wound	445	0.25
2	PC0646	Jul–2018	Yes	Street	Arm wound	445	0.19
2	PC0652	Mar–2019	Yes	Shelter D	Abdomen abscess	nt*	0.25
3	PC0648	Jan–2019	Yes	Shelter A	Blood	445	0.38
4	PC0649	Feb–2019	Yes	Shelters A, B	Leg wound	445	0.25
5	PC0651	Mar–2019	Yes	Shelter A	Buttock wound	445	0.25
6	PC0653	Mar–2019	Yes	Shelters A, C	Neck wound	445	0.38
7	PC0654	Apr–2019	No	na	Foot ulcer	445	0.19
8	PC0655	May–2019	Yes	Shelter B	Hand wound	445	0.38

*Single-locus variant of ST445 with synonymous SNP in *fusA.*

MIC, minimum inhibitory concentration (mg l^−1^); nt, new type; na, not applicable.

### Microbiological identification

Isolates were grown on trypticase soy agar with 5 % sheep blood (BBL, Sparks, MD, USA) and biochemically identified using API Coryne strips (bioMerieux, Durham, NC, USA). Toxin production was confirmed by Elek test [15] and antibiotic resistance to 11 different antibiotics (amoxicillin, azithromycin, clarithromycin, clindamycin, daptomycin, erythromycin, levofloxacin, meropenem, penicillin, rifampin and vancomycin) was determined by Etest (bioMerieux, Durham, NC, USA). Antibiotic resistance interpretative categories followed the Clinical and Laboratory Standard Institute (CLSI) 2015 guidelines, which for penicillin included ‘sensitive’ (MIC<=0.12 mg l^−1^), ‘intermediate’ (MIC>0.25–2.0 mg l^−1^), or resistant (MIC>=4.0 mg l^−1^). As clinical breakpoints for *Corynebacterium* species are not defined for amoxicillin, azithromycin, clarithromycin, or levofloxacin, these were not assigned an interpretive category.

### WGS

All *C. diphtheriae* isolates were grown on trypticase soy agar with 5 % sheep blood at 37 °C for 24 h. Genomic DNA was extracted using the Maxwell RSC Whole Blood DNA kit (Promega, San Luis Obispo, CA, USA). Genomic DNA for PacBio sequencing was further cleaned by salt/chloroform washing [16]. Genomic DNA concentrations were determined using the Qubit dsDNA broad range quantification kit (Thermo Fisher Scientific, Waltham, MA, USA). Paired-end libraries (2×250 bp) were prepared with the NEBNext Ultra DNA Library Prep kit (New England Biolabs, Ipswich, MA, USA) and sequenced on a Miseq using reagent v2, 500-cycle kit (Illumina, San Diego, CA, USA). The Illumina raw reads were checked for quality using FastQC v0.11.5 [17] and trimmed by Cutadapt [18]. Trimmed reads were *de novo* assembled using SPAdes v3.9 [19] and evaluated by QUAST v4.5 [20].

An additional PacBio library was prepared from PC0646 using the SMRTbell Template Prep kit 1.0 and Polymerase Binding kit P4. It was sequenced on the PacBio RSII sequencer (Pacific Biosciences, Menlo Park, CA, USA). *De novo* assembly was performed using the Hierarchical Genome Assembly Process (HGAP v3) (Pacific Biosciences, Menlo Park, CA, USA) [21].The resulting consensus sequence was manually checked for circularity using Gepard v1.30 [22] and further polished by mapping Illumina reads in CLC Genomics Workbench v12 (CLC Bio, Boston, MA, USA). The complete genome assembly of PC0646 was annotated using the National Center for Biotechnology Information (NCBI) Prokaryotic Genome Annotation Pipeline (PGAP).

### MLST and diphtheria toxin gene detection

MLST allele profiles were determined from the assembled contigs according to pubMLST [9], and further confirmed with trimmed sequencing reads using SRST2 [23]. The presence of diphtheria toxin homologues was determined by tblastn [24] query of the assembled genomes using select references: corynephage beta A and B subunits (NCBI accession number P00588), corynephage omega beta diphtheria toxin (accession number P00587), and corynephage beta diphtheria toxin homologue (accession number P00589).

Additionally, trimmed sequencing reads from all *C. diphtheriae* isolates were mapped to the reference genome of NCTC13129 (accession number NC_002935.2) using Snippy v4.3.8 with default settings [25] and coverage across the diphtheria toxin gene was calculated with SAMtools [26]. The detected presence or absence of the diphtheria toxin gene in each *C. diphtheriae* isolate is reported in Table S1 (available in the online version of this article).

### Whole-genome SNP phylogeny and characterization

Publicly available *C. diphtheriae* genomic data for 305 isolates was retrieved from the NCBI, including 284 raw Illumina sequencing read sets from SRA (downloaded on 22 November 2019) and 21 complete genomes (Table S2). Raw reads were trimmed and filtered as described above. SNPs were determined by mapping trimmed reads to the reference genome of NCTC13129 using Snippy v4.3.8 with default settings [25]. The resulting core SNP alignment was used to estimate the phylogeny using maximum-likelihood with RaxML v8.2.9 [27]. The final tree was visualized with iToL v4 [28]. Construction of additional subtrees, including for each of the clusters reported previously, followed a similar procedure using a reference genome selected from within each cluster. SNPs among the WA isolates were determined relative to the complete genome assembly of PC0646 (accession number CP040557) using Snippy and further characterized using SnpEff v4.3 [29]. Pairwise SNP distances were calculated from the subtree core alignments using SNP-Dist v4.0 [30].

### Virulence factor and resistance gene detection

The VF analyser from the Virulence Factors DataBase (VFDB) [31] was used to predict the virulence factor profiles of WA isolates using their genome assemblies. The predicted nucleotide sequences for *srtB*, *srtC* and *spaD* were extracted from genome assemblies of PC0653 and PC0654 for blastp [24] query against the NCBI non-redundant (nr) protein database. The unique region harbouring the above three genes was extracted from PC0653 and PC0654 and translated with GeneMark [32]. Additional protein-coding genes predicted by GeneMark were further blastp [24] queried in the NCBI nr protein database. In a similar manner, the presence of the novel penicillin binding protein encoding gene *pbp2m* [33] was also evaluated by querying the assembled contigs using blastn [24].

## Results

### Molecular characterization and antimicrobial susceptibility

Diagnostic microbiological assays identified all case isolates as nontoxigenic *C. diphtheriae* biovar mitis with intermediate resistance to penicillin (Table 1). The isolates were susceptible to 10 additional antibiotics and the *pbp2m* beta-lactam resistance gene was not detected in any assembled genomes. MLST determined from either assembled genomes or trimmed reads indicated that nine isolates shared ST445 (*aptA*, 3; *dnaE*, 2; *dnaK*, 60; *fusA*, 30; *leuA*, 3; *odhA*, 3; *rpoB*, 2), while PC0652 exhibited a single locus variant due to a synonymous SNP in *fusA,* whose sequence has been submitted to pubMLST and assigned as *fusA* 83.

### Core SNP phylogeny

**Fig. 1.**
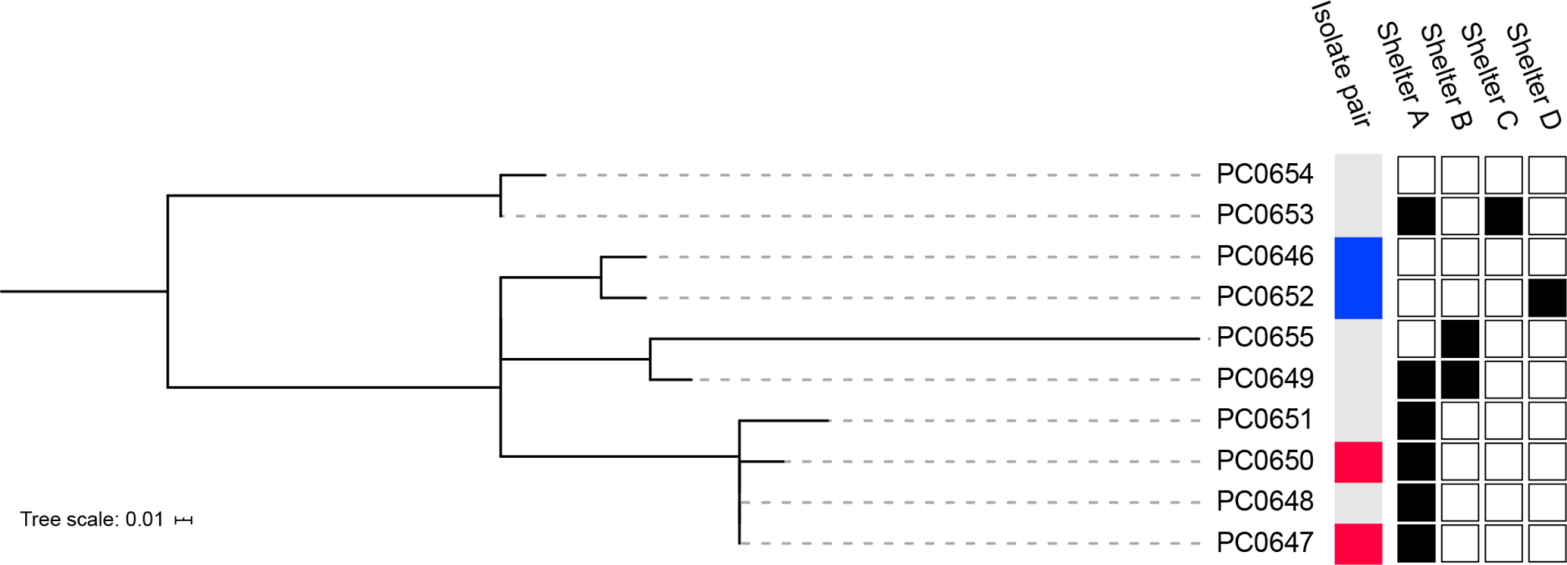
Phylogenetic reconstruction of the WA *C. diphtheriae* isolates from 37 core variable sites using maximum likelihood. Isolate pairs recovered from the same patient and visits to specific shelter locations are indicated next to the tree.

The phylogeny of WA *C. diphtheriae* isolates was reconstructed from 37 core SNPs to investigate their genetic relatedness using the complete genome of PC0646 as a reference (Fig. 1). The average pairwise distance between isolates was 11.6 SNPs (range: 0–24) (Table S3), consistent with other reported outbreaks of *C. diphtheriae* (Table S4). Isolates from three patients (PC0647, PC0648, PC0650, PC0651), all of whom attended shelter A, formed a tight cluster and differed from each other by an average of 1.5 SNPs (range: 0–3) (Fig. 1). Pairs of isolates recovered from individual patients (PC0646 and PC0652, PC0647 and PC0650) clustered together. In total, 51 unique mutations were detected among the WA isolates, 35 of which appeared in protein-coding genes (12 synonymous, 17 nonsynonymous and 6 indels) of various predicted functions that did not include known virulence factors (Table S5). Only a single SNP was observed between PC0647 and PC0650, a missense mutation in a gene encoding a predicted cell surface protein with high sequence similarity to substrate-binding components of ABC transporters. PC0646 and PC0652 differed by 2 SNPs, a synonymous substitution in *fusA* and an intergenic substitution upstream of an AraC family transcriptional regulator (Table 2).

**Table 2.** Single-nucleotide polymorphism annotations for the WA *C. diphtheriae* isolates from the same patient

Position*	Ref	Alt	Type	Gene	Amino acids	Function
PC0652 vs PC0646						
370 633	C	T	synonymous_variant	*fusA*	p.Ser314Ser	elongation factor G
808 375	T	C	upstream_gene_variant	FGA20_04195		AraC family transcriptional regulator
PC0647 vs PC0650						
2 323 370	G	T	missense_variant	FGA20_11260	p.Leu508Met	Cell surface protein

*Position in complete assembly of PC0646 (accession number CP040557).

Phylogenetic placement of the WA isolates was further investigated within the context of all publicly available *C. diphtheriae* genomic data from 305 isolates, including reported outbreak clusters from Switzerland and other geographically defined clusters (Fig. S1, Table 1). Among the publicly available genomic data, FRC0157 (ST367) was the most closely related to the WA isolates but still differed by more than 12 000 SNPs.

### Virulence factor profiles

All WA isolates were nontoxigenic as determined by the Elek test. The absence of the toxin-encoding corynebacteriophage was also confirmed by the genomic data. Genome sequence queries with a collection of known virulence factors detected the presence of 23 putative matches, most of which were conserved among all the WA isolates as well as nontoxigenic Canadian isolate CD10 (Table 3). Eight isolates (PC0646, PC0647, PC0648, PC0649, PC0650, PC0651, PC0652 and PC0655) shared an identical complement of virulence genes. Compared to VFDB reference NCTC13129, the WA isolate genomes encoded the same complement of iron and haem acquisition systems, but only one intact pilus locus (*spaABC*) was identified (Table 3). However, additional virulence genes (*srtB*, *srtC* and *spaD*) were detected in PC0653 and PC0654. These three genes were encoded within an 8.7 kb island that was absent from the complete genome assembly of PC0646. Compared to corresponding orthologues in NCTC13129, the relative sequence identity was 58 % for SrtB, 63 % SrtC and 41 % for SpaD. This 8.7 kb region was observed in the middle of a long contig (>25 kb) that had a high coverage (>100×), indicating that it was not an assembly artefact, and exhibited very high similarity (99 % identity) to *C. diphtheriae* isolate FRC0435 (ST411). Further blastp query of additional proteins encoded within the 8.7 kb region to the NCBI nr database revealed two possible matches to cell surface/membrane proteins found in *C. diphtheriae *, which had no homology to SpaE and SpaF (Table S6).

**Table 3.** Predicated virulence factor profiles for the WA isolates and reference genomes

Virulence factors	Genes	VFDB ID	NCTC13129* (Locus ID)	CD10†	PC0653 PC0654	PC0646‡
SpaA-type pili	*spaA*	VFG002201	DIP_RS21255	Y	Y	Y
	*spaB*	VFG002200	DIP_RS21245	Y	Y	Y
	*spaC*	VFG002199	DIP_RS21240	Y	Y	Y
	*srtA*	VFG013668	DIP_RS21250	Y	Y	Y
SpaD-type pili	*spaD*	VFG002202	DIP_RS12565	–	Y	–
	*spaE*	VFG002203	DIP_RS12575	–	–	–
	*spaF*	VFG002204	DIP_RS12580	–	–	–
	*srtB*	VFG013672	DIP_RS12555	Y	Y	–
	*srtC*	VFG013676	DIP_RS12570	Y	Y	–
SpaH-type pili	*spaG*	VFG002207	DIP_RS22255	–	–	–
	*spaH*	VFG002206	DIP_RS22250	–	–	–
	*spaI*	VFG002205	DIP_RS22235	–	–	–
	*srtD*	VFG013685	DIP_RS22245	–	–	–
	*srtE*	VFG013682	DIP_RS22240	–	–	–
Surface-anchored pilus proteins	*sapA*	VFG013691	DIP_RS21475	–	–	–
	*sapD*	VFG013693	DIP_RS13555	Y	Y	Y
	*sapE*	VFG013695	–	–	–	–
ABC transporter	*fagA*	VFG013742	DIP_RS16495	Y	Y	Y
	*fagB*	VFG013736	DIP_RS16490	Y	Y	Y
	*fagC*	VFG013730	DIP_RS16485	Y	Y	Y
	*fagD*	VFG013748	DIP_RS16500	Y	Y	Y
ABC-type haem transporter	*hmuT*	VFG013704	DIP_RS14430	Y	Y	Y
	*hmuU*	VFG013709	DIP_RS14435	Y	Y	Y
	*hmuV*	VFG013715	DIP_RS14440	Y	Y	Y
Siderophore-dependent iron uptake system	*irp6A*	VFG013698	DIP_RS11960	Y	Y	Y
	*irp6B*	VFG013700	DIP_RS11965	Y	Y	Y
	*irp6C*	VFG013702	DIP_RS11970	Y	Y	Y
ciu iron uptake and siderophore biosynthesis system	*ciuA*	VFG013721	DIP_RS14220	Y	Y	Y
	*ciuB*	VFG013723	DIP_RS14225	Y	Y	Y
	*ciuC*	VFG013725	DIP_RS14230	Y	Y	Y
	*ciuD*	VFG013727	DIP_RS14235	Y	Y	Y
	*ciuE*	VFG013729	DIP_RS14240	Y	Y	Y
Diphtheria toxin repressor DtxR	*dtxR*	VFG013754	DIP_RS18250	Y	Y	Y
Diphtheria toxin (DT)	*tox*	VFG002198	DIP_RS12515	–	–	–

*The NCBI accession number for NCTC13129 is NC002935.2.

†Core virulence factor profile for nontoxigenic *C. diphtheriae* ST8 circulating in Canada [13].

‡Seven WA isolates (PC0647, PC0648, PC0649, PC0650, PC0651, PC0652 and PC0655) share the same virulence factor profiles with PC0646.

## Discussion

To better understand the molecular diversity of nontoxigenic *C. diphtheriae* cutaneous infections, we performed WGS with 10 *C *. *diphtheriae* case isolates recovered from patients in King County, WA, USA to study their phylogeny and virulence factor profiles. The phylogenetic reconstruction showed that all WA isolates were closely related, but quite different from other sequenced isolates of *C. diphtheriae *. This indicated that these cutaneous infections were caused by a single cluster of nontoxigenic *C. diphtheriae *, which had accumulated enough mutations to resolve linkages between cases with shared epidemiology. We observed very few SNPs among pairs of isolates when recovered from the same patient, including those from different body sites, suggesting both patients likely suffered from a single, persistent infection rather than repeated infections.

Although all WA isolates are phylogenetically related, the genomes of two isolates (PC0653 and PC0654) possessed an additional region encoding more virulence factors (SrtB, SrtC and SpaD). Such virulence factor content variation among isolates of the same ST has been reported previously [13]. SrtB, SrtC and SpaD belong to the SpaD-like pilus gene cluster (*srtB-spaD-srtC-spaE-spaF*) and are essential for sortase-mediated pilus assembly, which mediates bacterial attachment and colonization of host tissues [34]. Similar pilus gene clusters are often reported on horizontally acquired genomic islands, and the number and organization of SpaD-like pilus gene clusters vary [35]. The gene content and organization of the region detected in PC0653 and PC0654 differed from previously reported SpaD-like pilus gene clusters [35, 36], suggesting that it may encode either a novel SpaD-like pilus or nonfunctional relic. Varied pilus gene cluster content is common among *C. diphtheriae* isolates and while it appears to alter macromolecular surface composition and influence adherence to specific host cell types, correlation with pathogenicity or invasion remains unclear [35–38]. The WA isolate genomes otherwise encoded the full complement of virulence genes in NCTC13129, specifically iron and haem acquisition systems essential for colonizing the low-iron host environment [39–42], corroborating their capacity for disease and concern of serious respiratory infection should they gain toxin-encoding bacteriophage. Regardless, these data illustrate that the *C. diphtheriae* genome is dynamic, varying in gene content even during transmission within a well-defined outbreak where pairwise SNP distance remains very low.

Previous reports from Canada, Poland and Germany have also investigated the circulation of nontoxigenic *C. diphtheriae* in urban impoverished populations, each involving a unique predominant ST primarily colonizing skin ulcerations [3, 6, 12, 13]. In addition, Lowe *et al.* observed that certain patients’ skin ulcerations developed into severe non-respiratory diseases including bacteraemia and endocarditis [3]. Poor hygiene conditions, homelessness, drug use and alcoholism were also identified as risk factors. Similarly, 7/8 (90 %) of the WA patients in this study experienced homelessness and most were documented drug users. Reduced susceptibility to first-line antimicrobials for treatment could further complicate persistent infections in this population, but a recent study suggests that breakpoints defining the intermediate penicillin resistance observed in isolates here may lack clinical importance [43]. As skin lesions may facilitate efficient transmission, particularly when coupled with poor hygiene*,* there is a need for increased awareness of the risk for persistent cutaneous infections of nontoxigenic *C. diphtheriae* in homeless populations.

## Supplementary Data

Supplementary material 1
