# Supplementary material for: Genomic epidemiology of nontoxigenic Corynebacterium diphtheriae from King County, Washington State, USA between July 2018 and May 2019
Source: Microb Genom. 2020 Dec 4;6(12):mgen000467. doi: 10.1099/mgen.0.000467 (PMC8116682; doi:10.1099/mgen.0.000467)
Supplement: Supplementary material 1 [file mgen-6-467-s001.pdf]

## Supplemental Material

**Figure S1**: Phylogenetic reconstruction of the WA *C. diphtheriae* isolates and additional publicly available genomic data from 305 isolates using 136180 core, variable sites with maximum likelihood. The cluster of WA isolates is indicated in red. Previously reported clusters from Switzerland (green) *[1]*, Canada (blue) *[2]*, and Germany (orange) *[3]* are indicated with their toxigenicity and isolate count. Details for additional geographically defined clusters (purple) are listed in Table S4.

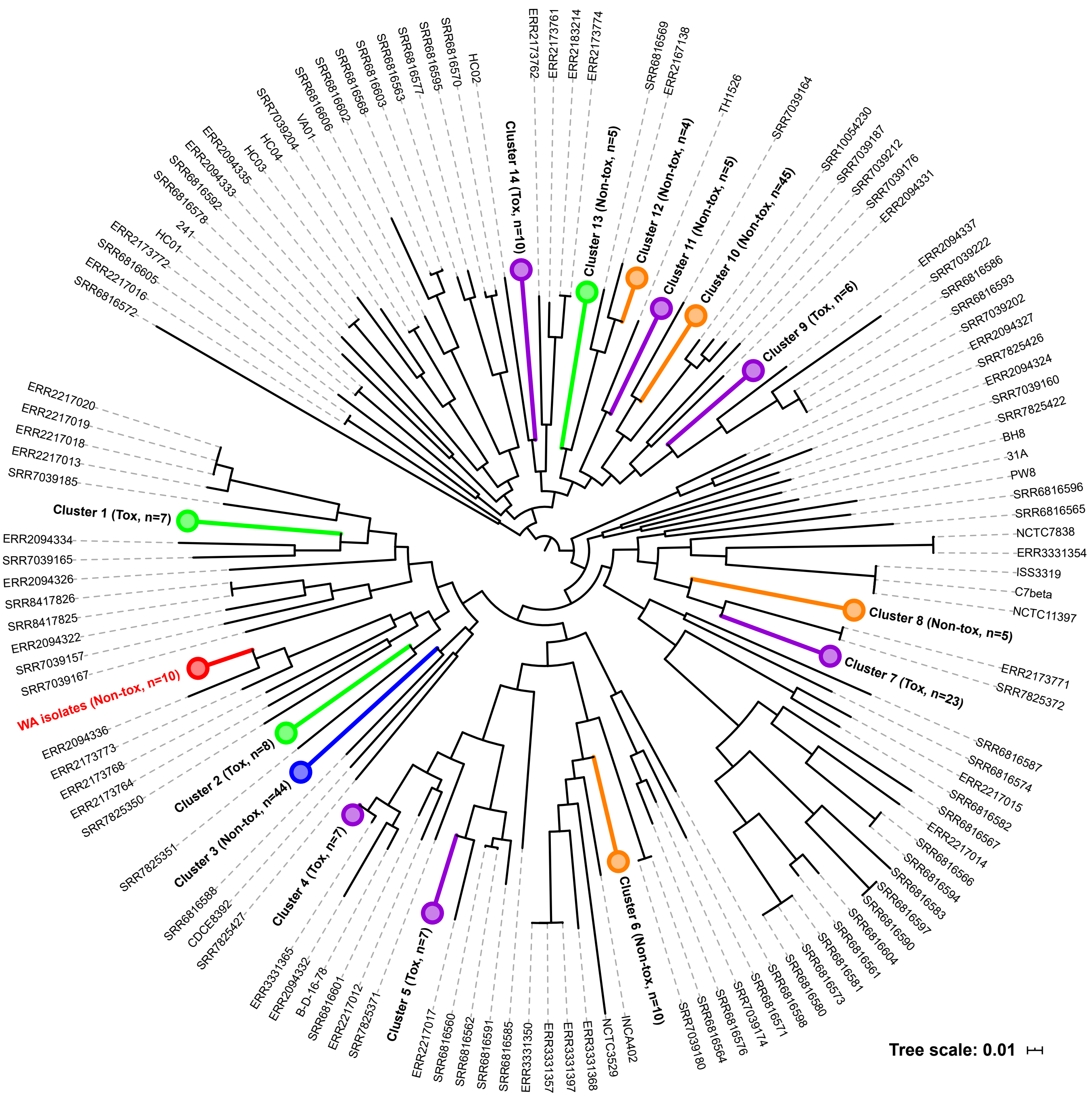

Cluster 1 (Tox, n=7)
Cluster 2 (Tox, n=8)
Cluster 3 (Non-tox, n=44)
Cluster 4 (Tox, n=7)
Cluster 5 (Tox, n=7)
Cluster 6 (Non-tox, n=10)
Cluster 7 (Tox, n=23)
Cluster 8 (Non-tox, n=5)
Cluster 9 (Tox, n=6)
Cluster 10 (Non-tox, n=45)
Cluster 11 (Non-tox, n=5)
Cluster 12 (Non-tox, n=4)
Cluster 13 (Non-tox, n=5)
Cluster 14 (Tox, n=10)
WA isolates (Non-tox, n=10)
Tree scale: 0.01
ERR2217020
ERR2217019
ERR2217018
ERR2217013
SRR7039185
ERR2094334
SRR7039165
ERR2094326
SRR8417826
SRR8417825
ERR2094322
SRR7039157
SRR7039167
ERR2094336
ERR2173773
ERR2173768
ERR2173764
SRR7825350
SRR7825351
SRR6816588
CDCE8392
SRR7825427
ERR3331365
ERR2094332
B-D-16-78
SRR6816601
ERR2217012
SRR7825371
ERR2217017
SRR6816560
SRR6816562
SRR6816591
SRR6816585
ERR3331350
ERR3331357
ERR3331397
ERR3331368
NCTC3529
INCA402
SRR7039180
SRR6816564
SRR6816576
SRR7039174
SRR6816571
SRR6816598
SRR6816580
SRR6816573
SRR6816581
SRR6816561
SRR6816604
SRR6816590
SRR6816597
SRR6816583
SRR6816594
SRR6816566
ERR2217014
SRR6816567
SRR6816582
ERR2217015
SRR6816574
SRR6816587
SRR7825372
ERR2173771
NCTC11397
C7beta
ISS3319
ERR3331354
NCTC7838
SRR6816565
SRR6816596
PW8
31A
BH8
SRR7825422
SRR7039160
ERR2094324
SRR7825426
ERR2094327
SRR7039202
SRR6816593
SRR6816586
SRR7039222
ERR2094337
ERR2094331
SRR7039176
SRR7039212
SRR7039187
SRR10054230
SRR7039164
TH1526
ERR2167138
SRR6816569
ERR2173774
ERR2183214
ERR2173761
ERR2173762
HC02
SRR6816570
SRR6816595
SRR6816577
SRR6816563
SRR6816603
SRR6816568
SRR6816602
SRR6816606
VA01
SRR7039204
HC04
HC03
ERR2094335
ERR2094333
SRR6816592
SRR6816578
241
HC01
ERR2173772
SRR6816605
ERR2217016
SRR6816572

**Table S1:** *C. diphtheriae* isolates within clustered highlighted in Figure S1.

| Cluster | Run | Alias Name | ST | Toxin gene presence (Yes/No) | Project | Country |
|---|---|---|---|---|---|---|
| WA | SRR12270040 | PC0646 | 445 | N | PRJNA541849 | USA |
| WA | SRR12270039 | PC0647 | 445 | N | PRJNA541849 | USA |
| WA | SRR12270038 | PC0648 | 445 | N | PRJNA541849 | USA |
| WA | SRR12270037 | PC0649 | 445 | N | PRJNA541849 | USA |
| WA | SRR12270036 | PC0650 | 445 | N | PRJNA541849 | USA |
| WA | SRR12270035 | PC0651 | 445 | N | PRJNA541849 | USA |
| WA | SRR12270034 | PC0652 | NT* | N | PRJNA541849 | USA |
| WA | SRR12270033 | PC0653 | 445 | N | PRJNA541849 | USA |
| WA | SRR12270032 | PC0654 | 445 | N | PRJNA541849 | USA |
| WA | SRR12270031 | PC0655 | 445 | N | PRJNA541849 | USA |
| 1 | ERR2173765 | Cd_6 | 486 | Y | PRJNA541849 | Switzerland |
| 1 | ERR2173769 | Cd_18 | 486 | Y | PRJEB14914 | Switzerland |
| 1 | ERR2173770 | Cd_19 | 486 | Y | PRJEB14914 | Switzerland |
| 1 | SRR7825355 | KL0652 | 486 | Y | PRJNA490531 | Germany |
| 1 | SRR7825367 | KL0613 | 486 | Y | PRJNA490531 | Germany |
| 1 | SRR7825368 | KL0625 | 486 | Y | PRJNA490531 | Germany |
| 1 | SRR7825369 | KL0623 | 486 | Y | PRJNA490531 | Germany |
| 2 | ERR2173763 | Cd_3 | 473 | Y | PRJEB14914 | Switzerland |
| 2 | ERR2173767 | Cd_9 | 473 | Y | PRJEB14914 | Switzerland |
| 2 | ERR2173855 | Cd-11 | 473 | Y | PRJEB14914 | Switzerland |
| 2 | SRR7825356 | KL0654 | 473 | Y | PRJNA490531 | Germany |
| 2 | SRR7825358 | KL0663 | 473 | Y | PRJNA490531 | Germany |
| 2 | SRR7825359 | KL0670 | 473 | Y | PRJNA490531 | Germany |
| 2 | SRR7825364 | KL0633 | 473 | Y | PRJNA490531 | Germany |
| 2 | SRR7825365 | KL0631 | 473 | Y | PRJNA490531 | Germany |
| 3 | SRR10054201 | CD35 | 76 | N | PRJNA563223 | Canada |
| 3 | SRR10054202 | CD36 | 76 | N | PRJNA563223 | Canada |
| 3 | SRR10054203 | CD37 | 76 | N | PRJNA563223 | Canada |
| 3 | SRR10054204 | CD38 | 76 | N | PRJNA563223 | Canada |
| 3 | SRR10054211 | CD44 | 76 | N | PRJNA563223 | Canada |
| 3 | SRR10054217 | CD50 | 76 | N | PRJNA563223 | Canada |
| 3 | SRR10054218 | CD6 | 76 | N | PRJNA563223 | Canada |
| 3 | SRR10054219 | CD51 | 76 | N | PRJNA563223 | Canada |
| 3 | SRR10054220 | CD52 | 76 | N | PRJNA563223 | Canada |
| 3 | SRR10054221 | CD53 | 76 | N | PRJNA563223 | Canada |
| 3 | SRR10054222 | CD54 | 76 | N | PRJNA563223 | Canada |
| 3 | SRR10054223 | CD55 | 76 | N | PRJNA563223 | Canada |
| 3 | SRR10054224 | CD56 | 76 | N | PRJNA563223 | Canada |
| 3 | SRR10054225 | CD7 | 76 | N | PRJNA563223 | Canada |
| 3 | SRR10054226 | CD8 | 76 | N | PRJNA563223 | Canada |
| 3 | SRR10054227 | CD9 | 76 | N | PRJNA563223 | Canada |
| 3 | SRR10054228 | CD10 | 76 | N | PRJNA563223 | Canada |
| 3 | SRR10054229 | CD34 | 76 | N | PRJNA563223 | Canada |
| 3 | SRR10054231 | CD32 | 76 | N | PRJNA563223 | Canada |
| 3 | SRR10054232 | CD31 | 76 | N | PRJNA563223 | Canada |
| 3 | SRR10054233 | CD4 | 76 | N | PRJNA563223 | Canada |
| 3 | SRR10054234 | CD30 | 76 | N | PRJNA563223 | Canada |
| 3 | SRR10054235 | CD29 | 76 | N | PRJNA563223 | Canada |
| 3 | SRR10054236 | CD28 | 76 | N | PRJNA563223 | Canada |
| 3 | SRR10054237 | CD27 | 76 | N | PRJNA563223 | Canada |
| 3 | SRR10054238 | CD26 | 76 | N | PRJNA563223 | Canada |
| 3 | SRR10054239 | CD25 | 76 | N | PRJNA563223 | Canada |
| 3 | SRR10054240 | CD24 | 76 | N | PRJNA563223 | Canada |
| 3 | SRR10054241 | CD23 | 76 | N | PRJNA563223 | Canada |
| 3 | SRR10054242 | CD22 | 76 | N | PRJNA563223 | Canada |
| 3 | SRR10054243 | CD21 | 76 | N | PRJNA563223 | Canada |
| 3 | SRR10054244 | CD3 | 76 | N | PRJNA563223 | Canada |
| 3 | SRR10054245 | CD20 | 76 | N | PRJNA563223 | Canada |
| 3 | SRR10054246 | CD19 | 76 | N | PRJNA563223 | Canada |
| 3 | SRR10054247 | CD18 | 76 | N | PRJNA563223 | Canada |
| 3 | SRR10054248 | CD17 | 76 | N | PRJNA563223 | Canada |
| 3 | SRR10054249 | CD16 | 76 | N | PRJNA563223 | Canada |
| 3 | SRR10054250 | CD15 | 76 | N | PRJNA563223 | Canada |
| 3 | SRR10054251 | CD14 | 76 | N | PRJNA563223 | Canada |
| 3 | SRR10054252 | CD13 | 76 | N | PRJNA563223 | Canada |
| 3 | SRR10054253 | CD12 | 76 | N | PRJNA563223 | Canada |

| | | | | | | |
|---|---|---|---|---|---|---|
| 3 | SRR10054254 | CD11 | 76 | N | PRJNA563223 | Canada |
| 3 | SRR10054255 | CD2 | 76 | N | PRJNA563223 | Canada |
| 3 | SRR10054256 | CD1 | 76 | N | PRJNA563223 | Canada |
| 4 | ERR3331362 | SAMEA5615640 | 209 | Y | PRJEB32654 | UK and vietanam |
| 4 | ERR3331366 | SAMEA5615644 | 209 | Y | PRJEB32654 | UK and vietanam |
| 4 | ERR3331372 | SAMEA5615650 | 209 | Y | PRJEB32654 | UK and vietanam |
| 4 | ERR3331384 | SAMEA5615662 | 209 | Y | PRJEB32654 | UK and vietanam |
| 4 | ERR3331386 | SAMEA5615664 | 209 | Y | PRJEB32654 | UK and vietanam |
| 4 | ERR3331387 | SAMEA5615665 | 209 | Y | PRJEB32654 | UK and vietanam |
| 4 | ERR3331395 | SAMEA5615674 | 209 | Y | PRJEB32654 | UK and vietanam |
| 5 | BQ11 | NA | 125 | Y | PRJNA224116 | Australia |
| 5 | SRR7223801 | Cdiphth_UnIn-R1 | 125 | Y | PRJNA473263 | Australia |
| 5 | SRR7223802 | Cdiphth_UnIn-R2 | 125 | Y | PRJNA473263 | Australia |
| 5 | SRR7223803 | Cdiphth_UnIn-R3 | 125 | Y | PRJNA473263 | Australia |
| 5 | SRR7223805 | Cdiphth_In-R1 | 125 | Y | PRJNA473263 | Australia |
| 5 | SRR7223806 | Cdiphth_In-R2 | 125 | Y | PRJNA473263 | Australia |
| 5 | SRR7223807 | Cdiphth_In-R3 | 125 | Y | PRJNA473263 | Australia |
| 6 | SRR7039158 | KL0371 | 130 | N | PRJNA416260 | Germany |
| 6 | SRR7039159 | KL0330 | 130 | N | PRJNA416260 | Germany |
| 6 | SRR7039162 | KL0438 | 130 | N | PRJNA416260 | Germany |
| 6 | SRR7039163 | KL0372 | 130 | N | PRJNA416260 | Germany |
| 6 | SRR7039178 | KL0507 | 130 | N | PRJNA416260 | Germany |
| 6 | SRR7039208 | KL0939 | 130 | N | PRJNA416260 | Germany |
| 6 | SRR7039210 | KL0937 | 130 | N | PRJNA416260 | Germany |
| 6 | SRR7039216 | KL0660 | 130 | N | PRJNA416260 | Germany |
| 6 | SRR7039221 | KL0943 | 130 | N | PRJNA416260 | Germany |
| 6 | SRR7039228 | KL0932 | 130 | N | PRJNA416260 | Germany |
| 7 | ERR3331348 | SAMEA5615626 | 67 | Y | PRJEB32654 | UK and vietanam |
| 7 | ERR3331349 | SAMEA5615627 | 67 | Y | PRJEB32654 | UK and vietanam |
| 7 | ERR3331351 | SAMEA5615629 | 67 | Y | PRJEB32654 | UK and vietanam |
| 7 | ERR3331353 | SAMEA5615631 | 67 | Y | PRJEB32654 | UK and vietanam |
| 7 | ERR3331355 | SAMEA5615633 | 67 | Y | PRJEB32654 | UK and vietanam |
| 7 | ERR3331358 | SAMEA5615636 | 67 | Y | PRJEB32654 | UK and vietanam |
| 7 | ERR3331359 | SAMEA5615637 | 67 | Y | PRJEB32654 | UK and vietanam |
| 7 | ERR3331360 | SAMEA5615638 | 67 | Y | PRJEB32654 | UK and vietanam |
| 7 | ERR3331363 | SAMEA5615641 | 67 | Y | PRJEB32654 | UK and vietanam |
| 7 | ERR3331370 | SAMEA5615648 | 67 | Y | PRJEB32654 | UK and vietanam |
| 7 | ERR3331375 | SAMEA5615653 | 67 | Y | PRJEB32654 | UK and vietanam |
| 7 | ERR3331376 | SAMEA5615654 | 67 | Y | PRJEB32654 | UK and vietanam |
| 7 | ERR3331377 | SAMEA5615655 | 67 | Y | PRJEB32654 | UK and vietanam |
| 7 | ERR3331378 | SAMEA5615656 | 67 | Y | PRJEB32654 | UK and vietanam |
| 7 | ERR3331382 | SAMEA5615660 | 67 | Y | PRJEB32654 | UK and vietanam |
| 7 | ERR3331388 | SAMEA5615666 | 67 | Y | PRJEB32654 | UK and vietanam |
| 7 | ERR3331389 | SAMEA5615667 | 67 | Y | PRJEB32654 | UK and vietanam |
| 7 | ERR3331391 | SAMEA5615670 | 67 | Y | PRJEB32654 | UK and vietanam |
| 7 | ERR3331392 | SAMEA5615671 | 67 | Y | PRJEB32654 | UK and vietanam |
| 7 | ERR3331393 | SAMEA5615672 | 67 | Y | PRJEB32654 | UK and vietanam |
| 7 | ERR3331394 | SAMEA5615673 | 67 | Y | PRJEB32654 | UK and vietanam |
| 7 | ERR3331396 | SAMEA5615675 | 67 | Y | PRJEB32654 | UK and vietanam |
| 7 | ERR3331398 | SAMEA5615677 | 67 | Y | PRJEB32654 | UK and vietanam |
| 8 | SRR7039170 | KL0964 | 439 | N | PRJNA416260 | Germany |
| 8 | SRR7039175 | KL0522 | 439 | N | PRJNA416260 | Germany |
| 8 | SRR7039193 | KL0954 | 439 | N | PRJNA416260 | Germany |
| 8 | SRR7039206 | KL0947 | 439 | N | PRJNA416260 | Germany |
| 8 | SRR7039214 | KL0676 | 439 | N | PRJNA416260 | Germany |
| 9 | ERR3331390 | SAMEA5615669 | 455 | Y | PRJEB32654 | UK and vietanam |
| 9 | ERR3331352 | SAMEA5615630 | 455 | Y | PRJEB32654 | UK and vietanam |
| 9 | ERR3331371 | SAMEA5615649 | 455 | Y | PRJEB32654 | UK and vietanam |
| 9 | ERR3331373 | SAMEA5615651 | 455 | Y | PRJEB32654 | UK and vietanam |
| 9 | ERR3331381 | SAMEA5615659 | 455 | Y | PRJEB32654 | UK and vietanam |
| 9 | ERR3331399 | SAMEA5615678 | 455 | Y | PRJEB32654 | UK and vietanam |
| 10 | NCTC13129 | NA | 8 | Y | PRJNA224116 | UK |
| 10 | SRR4271515 | FDAARGOS 197 | 8 | Y | PRJNA231221 | USA |
| 10 | SRR7039153 | KL0811 | 8 | N | PRJNA490531 | Germany |
| 10 | SRR7039154 | KL0945 | 8 | N | PRJNA490531 | Germany |
| 10 | SRR7039156 | KL0812 | 8 | N | PRJNA490531 | Germany |
| 10 | SRR7039161 | KL0759 | 8 | N | PRJNA490531 | Germany |
| 10 | SRR7039166 | KL0788 | 8 | N | PRJNA490531 | Germany |
| 10 | SRR7039168 | KL0768 | 8 | N | PRJNA490531 | Germany |

| | | | | | | |
|---|---|---|---|---|---|---|
| 10 | SRR7039169 | KL0770 | 8 | N | PRJNA490531 | Germany |
| 10 | SRR7039171 | KL0649 | 8 | N | PRJNA490531 | Germany |
| 10 | SRR7039172 | KL0659 | 8 | N | PRJNA490531 | Germany |
| 10 | SRR7039173 | KL0813 | 8 | N | PRJNA490531 | Germany |
| 10 | SRR7039179 | KL0628 | 8 | N | PRJNA490531 | Germany |
| 10 | SRR7039181 | KL0585 | 8 | N | PRJNA490531 | Germany |
| 10 | SRR7039182 | KL0599 | 8 | N | PRJNA490531 | Germany |
| 10 | SRR7039183 | KL0854 | 8 | N | PRJNA490531 | Germany |
| 10 | SRR7039184 | KL0899 | 8 | N | PRJNA490531 | Germany |
| 10 | SRR7039186 | KL0891 | 8 | N | PRJNA490531 | Germany |
| 10 | SRR7039188 | KL0881 | 8 | N | PRJNA490531 | Germany |
| 10 | SRR7039189 | KL0848 | 8 | N | PRJNA490531 | Germany |
| 10 | SRR7039190 | KL0872 | 8 | N | PRJNA490531 | Germany |
| 10 | SRR7039191 | KL0935 | 8 | N | PRJNA490531 | Germany |
| 10 | SRR7039192 | KL0953 | 8 | N | PRJNA490531 | Germany |
| 10 | SRR7039195 | KL0833 | 8 | N | PRJNA490531 | Germany |
| 10 | SRR7039198 | KL0929 | 8 | N | PRJNA490531 | Germany |
| 10 | SRR7039199 | KL0823 | 8 | N | PRJNA490531 | Germany |
| 10 | SRR7039200 | KL0844 | 8 | N | PRJNA490531 | Germany |
| 10 | SRR7039201 | KL0847 | 8 | N | PRJNA490531 | Germany |
| 10 | SRR7039203 | KL0834 | 8 | N | PRJNA490531 | Germany |
| 10 | SRR7039205 | KL0798 | 8 | N | PRJNA490531 | Germany |
| 10 | SRR7039209 | KL0871 | 8 | N | PRJNA490531 | Germany |
| 10 | SRR7039211 | KL0845 | 8 | N | PRJNA490531 | Germany |
| 10 | SRR7039213 | KL0678 | 8 | N | PRJNA490531 | Germany |
| 10 | SRR7039215 | KL0675 | 8 | N | PRJNA490531 | Germany |
| 10 | SRR7039217 | KL0705 | 8 | N | PRJNA490531 | Germany |
| 10 | SRR7039218 | KL0698 | 8 | N | PRJNA490531 | Germany |
| 10 | SRR7039219 | KL0693 | 8 | N | PRJNA490531 | Germany |
| 10 | SRR7039220 | KL0691 | 8 | N | PRJNA490531 | Germany |
| 10 | SRR7039223 | KL0747 | 8 | N | PRJNA490531 | Germany |
| 10 | SRR7039224 | KL0713 | 8 | N | PRJNA490531 | Germany |
| 10 | SRR7039225 | KL0865 | 8 | N | PRJNA490531 | Germany |
| 10 | SRR7039226 | KL0858 | 8 | N | PRJNA490531 | Germany |
| 10 | SRR7039227 | KL0933 | 8 | N | PRJNA490531 | Germany |
| 10 | SRR7825441 | KL0927 | 8 | N | PRJNA490531 | Germany |
| 11 | SRR6816589 | SAMN08624601 | 32 | N | PRJNA436425 | Austrilia |
| 11 | SRR6816584 | SAMN08624601 | 32 | N | PRJNA436425 | Austrilia |
| 11 | SRR6816575 | SAMN08624601 | 32 | N | PRJNA436425 | Austrilia |
| 11 | SRR6816599 | SAMN08624601 | 32** | N | PRJNA436425 | Austrilia |
| 11 | SRR6816600 | SAMN08624601 | 32** | N | PRJNA436425 | Austrilia |
| 12 | SRR7039155 | KL0476 | 295 | N | PRJNA416260 | Germany |
| 12 | SRR7039194 | KL0961 | 509 | N | PRJNA416260 | Germany |
| 12 | SRR7039196 | KL0965 | 295 | N | PRJNA416260 | Germany |
| 12 | SRR7039207 | KL0895 | 295 | N | PRJNA416260 | Germany |
| 13 | ERR2173766 | Cd_8 | 398 | N | PRJEB14914 | Switzerland |
| 13 | ERR2173856 | Cd-15 | 398 | N | PRJEB14914 | Switzerland |
| 13 | ERR2173857 | Cd-16 | 398 | N | PRJEB14914 | Switzerland |
| 13 | ERR2173858 | Cd-17 | 398 | N | PRJEB14914 | Switzerland |
| 13 | SRR7825357 | KL0655 | 398 | N | PRJNA490531 | Germany |
| 14 | ERR3331346 | SAMEA5615624 | 258 | N | PRJEB32654 | UK and vietanam |
| 14 | ERR3331347 | SAMEA5615625 | 258 | N | PRJEB32654 | UK and vietanam |
| 14 | ERR3331361 | SAMEA5615639 | 258 | N | PRJEB32654 | UK and vietanam |
| 14 | ERR3331364 | SAMEA5615642 | 258 | N | PRJEB32654 | UK and vietanam |
| 14 | ERR3331369 | SAMEA5615647 | 258 | N | PRJEB32654 | UK and vietanam |
| 14 | ERR3331374 | SAMEA5615652 | 258 | N | PRJEB32654 | UK and vietanam |
| 14 | ERR3331379 | SAMEA5615657 | 258 | N | PRJEB32654 | UK and vietanam |
| 14 | ERR3331380 | SAMEA5615658 | 258 | N | PRJEB32654 | UK and vietanam |
| 14 | ERR3331383 | SAMEA5615661 | 258 | N | PRJEB32654 | UK and vietanam |
| 14 | ERR3331385 | SAMEA5615663 | 258 | N | PRJEB32654 | UK and vietanam |

NT: new type

* one SNP in *fusA*

** one SNP in *dnaK*

**Table S2:** Available complete genomes of *C. diphtheriae* isolates used in this study.

| Strain | MLST | Accession No. |
|---|---|---|
| NCTC11397 | ST26 | NZ_LN831026.1 |
| NCTC 13129 | ST8 | NC_002935.2 |
| 31A | ST584 | NC_016799.1 |
| 241 | ST175 | NC_016782.1 |
| INCA 402 | ST173 | NC_016783.1 |
| BH8 | ST172 | NC_016800.1 |
| HC03 | ST171 | NC_016787.1 |
| HC02 | ST176 | NC_016802.1 |
| C7 (beta) | ST26 | NC_016801.1 |
| HC04 | ST128 | NC_016788.1 |
| CDCE 8392/CD174 | ST50 | NC_016785.1 |
| HC01 | ST175 | NC_016786.1 |
| VA01 | ST80 | NC_016790.1 |
| PW8 | ST44 | NC_016789.1 |
| B-D-16-78 | ST453 | NZ_CP018331.1 |
| FDAARGOS_197 | ST8 | NZ_CP020410.2 |
| ISS 3319 | ST26 | NZ_CP025209.1 |
| BQ11 | ST125 | NZ_CP029644.1 |
| TH1526 | ST377 | CP038504.1 |
| NCTC3529 | ST452 | NZ_LR134538.1 |
| NCTC7838 | ST10 | NZ_LR134537.1 |

**Table S3:** Pairwise SNP distance among the WA *C. diphtheriae* isolates

| | **PC0646** | **PC0647** | **PC0648** | **PC0649** | **PC0650** | **PC0651** | **PC0652** | **PC0653** | **PC0654** | **PC0655** |
|---|---|---|---|---|---|---|---|---|---|---|
| **PC0646** | 0 | 8 | 8 | 7 | 9 | 10 | 2 | 14 | 15 | 15 |
| **PC0647** | 8 | 0 | 0 | 9 | 1 | 2 | 8 | 16 | 17 | 17 |
| **PC0648** | 8 | 0 | 0 | 9 | 1 | 2 | 8 | 16 | 17 | 17 |
| **PC0649** | 7 | 9 | 9 | 0 | 10 | 11 | 7 | 15 | 16 | 10 |
| **PC0650** | 9 | 1 | 1 | 10 | 0 | 3 | 9 | 17 | 18 | 18 |
| **PC0651** | 10 | 2 | 2 | 11 | 3 | 0 | 10 | 18 | 19 | 19 |
| **PC0652** | 2 | 8 | 8 | 7 | 9 | 10 | 0 | 14 | 15 | 15 |
| **PC0653** | 14 | 16 | 16 | 15 | 17 | 18 | 14 | 0 | 1 | 23 |
| **PC0654** | 15 | 17 | 17 | 16 | 18 | 19 | 15 | 1 | 0 | 24 |
| **PC0655** | 15 | 17 | 17 | 10 | 18 | 19 | 15 | 23 | 24 | 0 |

**Table S4**: Pairwise SNP distances for the WA isolates and other clusters in Figure S1

| Cluster[1] | No. of isolates | MLST | Reference | Average pairwise SNP distance and range | Country |
|---|---|---|---|---|---|
| WA | 10 | ST445/NT | PC0646 | 11.6 (0-24) | United States |
| 3 | 44 | ST76 | SRR10054211 | 19.2 (0-157) | Canada *[2]* |
| 4 | 7 | ST209 | ERR3331366 | 36.6 (0-51) | Vietnam and UK |
| 5 | 7 | ST125 | BQ11 | 0.6 (0-1) | Australia |
| 6 | 10 | ST130 | SRR7039158 | 36.3 (1-78) | Germany |
| 7 | 23 | ST67 | ERR3331348 | 144.3 (0-282) | Vietnam and UK |
| 8 | 5 | ST439 | SRR7039170 | 74.6 (10-119) | Germany |
| 9 | 6 | ST455 | ERR3331371 | 32.3 (15-52) | Vietnam and UK |
| 10 | 45 | ST8 | NCTC13129 | 63.8 (0-113) | Germany *[3]* |
| 11 | 5 | ST32 | SRR6816589 | 77.4 (0-98) | Australia |
| 12 | 4 | ST295 & 509 | SRR7039155 | 160.5 (101-186) | Germany |
| 14 | 10 | ST258 | ERR3331346 | 143.3 (4-363) | Vietnam and UK |

[1]cluster 1,2,13 contained isolates from Switzerland outbreak and they pairwise SNP distances were up to 150 SNPs *[1]*.

NT: new type

**Table S5**: Detected mutations among the WA C. diphtheriae isolates relative to the closed genome assembly of PC0646.

| **Position** | **Ref** | **Alt** | **Count** | **Mutation Type** | **Locus Tag** | **AA Change** | **Gene Annotation** | **PC0647** | **PC0648** | **PC0649** | **PC0650** | **PC0651** | **PC0652** | **PC0653** | **PC0654** | **PC0655** |
|---|---|---|---|---|---|---|---|---|---|---|---|---|---|---|---|---|
| 4708 | CG | C | 8 | CDS_frameshift | FGA20_00015 | p.Gly393fs | DNA replication/repair protein RecF | x | x | x | x | x | | x | x | x |
| 144412 | C | CG | 3 | Intergenic | | | | | x | | | x | | x | | |
| 175418 | C | T | 4 | CDS_nonsynonymous | FGA20_01015 | p.Leu94Phe | sugar-binding transcriptional regulator | x | x | | x | x | | | | |
| 208374 | A | G | 9 | CDS_synonymous | FGA20_01185 | p.Val336Val | penicillin-binding protein | x | x | x | x | x | x | x | x | x |
| 212404 | T | TG | 1 | Intergenic | | | | | | x | | | | | | |
| 219339 | AC | A | 3 | Intergenic | | | | | | x | | | | x | x | |
| 338731 | T | C | 2 | CDS_nonsynonymous | FGA20_01905 | p.Leu277Ser | ABC transporter permease | | | | | | | x | x | |
| 370633 | C | T | 1 | CDS_synonymous | FGA20_02030 | p.Ser314Ser | elongation factor G fusA | | | | | | x | | | |
| 385202 | TA | AC | 1 | Intergenic | | | | | | | | | | | | x |
| 397459 | A | G | 2 | CDS_nonsynonymous | FGA20_02185 | p.Val289Ala | nitrate reductase subunit beta narH | | | | | | | x | x | |
| 432888 | A | AT | 2 | CDS_frameshift | FGA20_02350 | p.Cys158fs | sugar ABC transporter permease | | | | | | | x | x | |
| 441663 | C | T | 1 | CDS_nonsynonymous | FGA20_02390 | p.Ala23Val | exo-alpha-sialidase | | | | | | | | | x |
| 519404 | GC | G | 8 | Intergenic | | | | x | | x | x | x | x | x | x | x |
| 546196 | C | CG | 9 | Intergenic | | | | x | x | x | x | x | x | x | x | x |
| 646360 | A | G | 2 | CDS_nonsynonymous | FGA20_03370 | p.Glu21Gly | biotin/lipoyl-binding protein | | | | | | | x | x | |
| 665317 | C | G | 8 | CDS_synonymous | FGA20_03465 | p.Arg45Arg | transposase | x | x | x | x | x | | x | x | x |
| 696524 | C | A | 2 | CDS_nonsynonymous | FGA20_03635 | p.Ala111Ser | phosphoserine transaminase serC | | | | | | | x | x | |
| 709710 | G | C | 1 | Intergenic | | | | | | | | | | | | x |
| 742909 | G | A | 1 | CDS_nonsynonymous | FGA20_03870 | p.Gly454Glu | HAMP domain-containing histidine kinase | | | | | x | | | | |
| 808375 | T | C | 9 | Intergenic | | | | x | x | x | x | x | x | x | x | x |
| 817405 | A | T | 2 | CDS_nonsynonymous | FGA20_04245 | p.Lys245Met | serine hydroxymethyltransferase | | | | | | | x | x | |
| 907208 | C | T | 2 | CDS_synonymous | FGA20_04680 | p.Asp207Asp | DEAD/DEAH box helicase | | | | | | | x | x | |
| 943722 | C | T | 1 | CDS_synonymous | FGA20_04835 | p.Ser235Ser | F0F1 ATP synthase subunit delta | | | | | | | | x | |
| 959126 | A | G | 1 | CDS_synonymous | FGA20_04930 | p.Tyr676Tyr | DUF3416 domain-containing protein | | | | | | | | | x |
| 1023707 | AC | A | 1 | Intergenic | | | | | x | | | | | | | |
| 1023707 | ACCC | A | 1 | Intergenic | | | | | | | | | | | x | |
| 1106552 | G | A | 2 | CDS_nonsynonymous | FGA20_05605 | p.Val1Met | dethiobiotin synthetase | | | | | | | x | x | |
| 1164411 | G | T | 2 | CDS_nonsynonymous | FGA20_05870 | p.Ser13Ile | tRNA (cytidine(34)-2'-O)-methyltransferase | | | | | | | x | x | |
| 1171833 | A | AT | 1 | CDS_frameshift | FGA20_05910 | p.Cys447fs | proteasome ATPase arc | | | | | x | | | | |

| | | | | | | | | | | | | | | | | |
|---|---|---|---|---|---|---|---|---|---|---|---|---|---|---|---|---|
| 1219451 | C | T | 4 | CDS_synonymous | FGA20_06115 | p.Leu135Leu | metal-sulfur cluster assembly factor | x | x | | x | x | | | | |
| 1408510 | A | G | 2 | CDS_synonymous | FGA20_07025 | p.Cys82Cys | CinA family protein | | | | | | | x | x | |
| 1499299 | G | A | 1 | Intergenic | | | | | | | | | | | x | |
| 1506899 | T | G | 1 | CDS_nonsynonymous | FGA20_07460 | p.Lys405Thr | transposase family protein | | | | | | | | x | |
| 1650645 | T | A | 2 | CDS_nonsynonymous | FGA20_08120 | p.Tyr234Phe | alpha/beta hydrolase | | | | | | | x | x | |
| 1650923 | T | C | 1 | CDS_synonymous | FGA20_08120 | p.Glu141Glu | alpha/beta hydrolase | | | | | x | | | | |
| 1663026 | GA | G | 2 | CDS_frameshift | FGA20_08200 | p.Asp148fs | TPM domain-containing protein | | | | | | | x | x | |
| 1691345 | T | C | 1 | CDS_nonsynonymous | FGA20_08340 | p.Ile349Val | HNH endonuclease | | | | | | | | | x |
| 1692391 | T | C | 2 | Intergenic | | | | | | x | | | | | | x |
| 1758345 | G | GC | 2 | CDS_frameshift | FGA20_08650 | p.Pro65fs | DsbA family protein | | | x | | | | | | x |
| 1765503 | G | A | 4 | CDS_synonymous | FGA20_08680 | p.Ile106Ile | energy-dependent translational throttle protein ettA | x | x | | x | x | | | | |
| 1874191 | G | T | 1 | Intergenic | | | | | | | | | | | | x |
| 1877963 | C | A | 1 | Intergenic | | | | | x | | | | | | | |
| 1973285 | T | G | 2 | CDS_nonsynonymous | FGA20_09680 | p.Glu465Asp | isopeptide-forming domain-containing fimbrial | | | x | | | | | | x |
| 2027854 | T | C | 2 | Intergenic | | | | | | x | | | | | | x |
| 2060141 | G | A | 4 | CDS_nonsynonymous | FGA20_10060 | p.His5Tyr | hypothetical protein | x | x | | x | x | | | | |
| 2060161 | T | TG | 4 | CDS_frameshift | FGA20_10065 | p.Ile76fs | hypothetical protein | x | x | | x | x | | | | |
| 2060266 | T | A | 1 | CDS_synonymous | FGA20_10065 | p.Gly40Gly | hypothetical protein | | x | | | | | | | |
| 2060284 | T | C | 4 | CDS_synonymous | FGA20_10065 | p.Arg34Arg | hypothetical protein | x | x | | x | x | | | | |
| 2063716 | T | A | 2 | Intergenic | | | | | | | | | | x | x | |
| 2133313 | G | T | 1 | Intergenic | | | | | | x | | | | | | |
| 2323370 | G | T | 1 | CDS_nonsynonymous | FGA20_11260 | p.Leu508Met | Cell surface protein | | | | x | | | | | |

**Table S6**: BLASTp query results for the unique 8.7kb region detected in PC0653 and PC0654

| Nucleotide sequence position in the 8.7 kb region | VF analyzer prediction | BLASTp top hit (NCBI accession no.) | Amino acid sequence identity to isolate FRC0435 |
|---|---|---|---|
| 1257-442 bp | NA | Cell surface protein (CAB0925098.1) | 95% |
| 2195-1257 bp | SrtC | Class C sortase (CAB0925096.1) | 99% |
| 3893-2346 bp | SpaD | Isopeptide_forming domain-containing fimbriae protein (CAB0925093.1) | 99% |
| 4566-5504 bp | SrtB | Class C sortase (CAB0925087.1) | 100% |
| 6917-8710 bp | NA | Membrane protein (CAB0925084.1) | 100% |

NA: not applicable

References:

1. Meinel, D.M., et al., *Outbreak investigation for toxigenic Corynebacterium diphtheriae wound infections in refugees from Northeast Africa and Syria in Switzerland and Germany by whole genome sequencing.* Clin Microbiol Infect, 2016. **22**(12): p. 1003 e1-1003 e8.
2. Chorlton, S.D., et al., *Whole-Genome Sequencing of Corynebacterium diphtheriae Isolates Recovered from an Inner-City Population Demonstrates the Predominance of a Single Molecular Strain.* J Clin Microbiol, 2020. **58**(2).
3. Dangel, A., et al., *Geographically Diverse Clusters of Nontoxigenic Corynebacterium diphtheriae Infection, Germany, 2016-2017.* Emerg Infect Dis, 2018. **24**(7): p. 1239-1245.
